# Practical recommendations for diagnosis, management, and follow-up of Niemann-Pick type-C disease patients: a Brazilian perspective

**DOI:** 10.1055/s-0045-1807714

**Published:** 2025-05-09

**Authors:** Dafne Dain Gandelman Horovitz, André Pessoa, Marcondes Cavalcante França Junior, Roberto Giugliani, Carolina Fischinger Moura de Souza, Emília Katiane Embiruçu, Pedro Braga-Neto, Charles Marques Lourenço

**Affiliations:** 1 Fundação Oswaldo Cruz, Instituto Nacional de Saúde da Mulher, da Criança e do Adolescente Fernandes Figueira, Departamento de Genética Médica, Rio de Janeiro RJ, Brazil.; 2 Hospital Infantil Albert Sabin, Serviço de Neurologia e Neurogenética, Fortaleza CE, Brazil.; 3 Universidade Federal do Ceará, Faculdade de Medicina, Fortaleza CE, Brazil.; 4 Universidade Estadual de de Campinas, Faculdade de Ciências Médicas, Departamento de Neurologia, Campinas SP, Brazil.; 5 Universidade Federal do Rio Grande do Sul, Departamento de Genética Médica, Porto Alegre RS, Brazil.; 6 Dasa Genômica, Porto Alegre RS, Brazil.; 7 Casa dos Raros, Centro de Atenção Integral e Treinamento em Doenças Raras, Porto Alegre RS, Brazil.; 8 Hospital de Clínicas de Porto Alegre, Serviço de Genética Médica, Porto Alegre RS, Brazil.; 9 Universidade do Estado da Bahia, Departamento de Ciências da Vida, Salvador BA, Brazil.; 10 Hospital Universitário Professor Edgard Santos, Serviço de Genética Médica, Salvador BA, Brazil.; 11 Faculdade de Medicina de São José do Rio Preto, Centro de Referência de Doenças Raras, Unidade de Neurogenética, Clínica de Erros Inatos do Metabolismo, São José do Rio Preto SP, Brazil.

**Keywords:** Practice Guideline, Niemann-Pick Disease, Type C, Brazil, Lysosomal Storage Diseases

## Abstract

Niemann-Pick type-C (NPC) disease is a rare genetic condition with a clinical spectrum ranging from a fatal prenatally-presenting and quickly lethal disorder to an adult-onset chronic neurodegenerative condition. Given the scarcity of information regarding NPC disease in Brazil, a group of experts decided to discuss some disease-related aspects at the national level. The present manuscript describes the results of a Brazilian consensus meeting conducted to propose recommendations for the diagnosis, management, and follow-up of NPC disease in Brazil, considering the clinical practice point of view. These recommendations include patient characteristics on clinical presentation, as systemic and neurological manifestations according to the age group and atypical manifestations; a flowchart for diagnostic confirmation, considering the Brazilian scenario; and treatment, encompassing disease-modifying therapy, supportive care, and patients' follow-up. The expert panel provided an objective basis of recommendations on NPC diagnosis and management in Brazil. The authors expect that this manuscript will help clinicians to identify, adequately treat and follow-up NPC patients in Brazil.

## INTRODUCTION

Niemann-Pick type-C (NPC) disease is a rare genetic condition, caused by biallelic pathogenic variants in homozygosity or compound heterozygosity in any of two genes ( *NPC1* and *NPC2* ) that encode proteins involved in the intralysosomal cholesterol trafficking. The clinical spectrum ranges from a fatal prenatal disorder to an adult-onset, chronic, neurodegenerative disease. The rare prevalence of the disease and the lack of specialized care lead to misdiagnosis or late diagnosis, in addition to barriers to proper care. Such aspects contribute to physical, psychological, and intellectual impairments, resulting in major disability. 1

Niemann-Pick type-C disease is a rare condition with an estimated incidence of 1 case per 100,000 live births. The disease is pan-ethnic, and pathogenic variants in the *NPC1* gene cause at least 95% of all cases. 2 Burton et al. (2021) conducted a study to determine the disease prevalence in the United States and estimated 2.9 cases per million inhabitants. 3 The minimal incidence for Brazil was calculated as 0.304/100,000. 4

Niemann-Pick type-C disease has a complex physiopathology, starting from a simple failure of cholesterol export, progressing via multiple pathways to affect numerous cellular functions and results leading to early cellular death. This multi-faceted pathology poses a difficult challenge to develop therapies for this disorder. 5 To date, as it happens with other rare disorders, no specific curative therapy is available, and NPC disease usually progresses to premature death. Miglustat, a substrate-reducing therapy, is the only disease-modifying drug approved in Brazil for treating neurological manifestations, attenuating, or even stopping disease progression. However, the mainstay of treatment is symptomatic supportive therapy with a multidisciplinary team. 2

Recommendations on NPC diagnosis and management have been proposed by the international community since 2009, with two subsequent updates published. 6 7 8 9 The most recently published document proposes the use of supportive therapies in addition to miglustat for all patients with a confirmed diagnosis, except for those presymptomatic or presenting only an enlarged spleen or liver. In addition, diagnostic guidelines have been proposed. 8

Given the scarcity of information regarding NPC in Brazil, a group of experts was invited to discuss some disease-related aspects at the national level. The members of the group have followed approximately 35 patients with NPC disease, demonstrating the great experience of participants with this rare condition. This manuscript describes the results of a Brazilian consensus meeting conducted to propose recommendations for the diagnosis, management, and follow-up of NPC in Brazil, considering the clinical practice point of view.

## METHODS

On July 14, 2021, a 4-hour expert panel was virtually conducted (Zoom online platform link) to define national NPC diagnosis and management recommendations. Before the expert panel took place, an electronic questionnaire was sent to participants covering the following topics: general disease characteristics, clinical presentation, complementary exams, and treatment. The answers were compiled and analyzed to guide the discussion that served as a basis for this manuscript construction.

### Diagnosis

#### *Characteristics on clinical presentation*

The age of symptoms onset must be actively investigated because this is an important prognostic marker; the earlier the symptoms onset the worse the long-term prognosis. However, continuous observation of disease evolution is necessary. Figure 1 shows a graphic scheme proposed by Vanier et al. (2010) for clinical disease presentation according to age. 1

**Adapted from:Figure 1:**
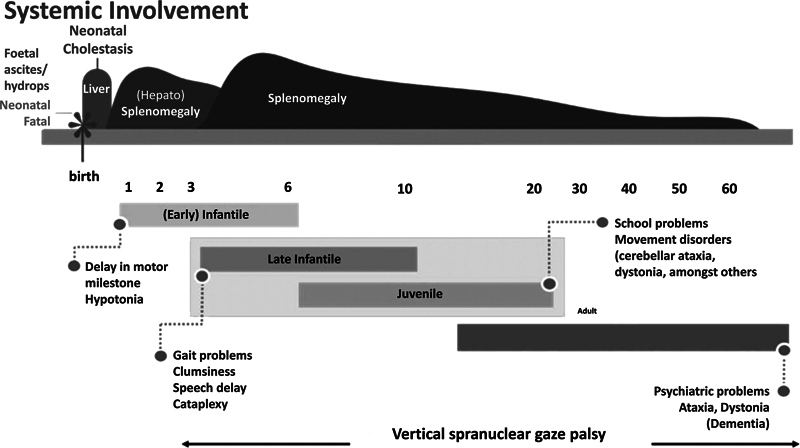
Vanier MT. Orphanet J Rare Dis. 2010 Jun 3;5:16. 1 Niemann-Pick Type-C clinical manifestations according to the age of onset.

In Table 1 , systemic and neurological manifestations to be observed in each age group are described, according to Geberhiwot et al. (2018). 8 Among the pre/perinatal most frequently observed manifestations are fetal ascites/hydrops, hepatosplenomegaly, prolonged neonatal jaundice/cholestasis, thrombocytopenia, pulmonary disease, liver failure, failure to thrive, and hypotonia. 8 10 Considering early-infantile patients, manifestations may include hepatosplenomegaly or splenomegaly (isolated or with neurological manifestations), prolonged neonatal jaundice/cholestasis, central hypotonia, delayed developmental motor milestones, resembling floppy infant syndrome, speech delay, dysphagia, spasticity, vertical supranuclear gaze palsy (VSGP), and ataxia. Manifestations observed among late-infantile patients may include hepatosplenomegaly or splenomegaly (isolated or with neurological manifestations), history of prolonged neonatal jaundice/cholestasis, developmental delay/regression, speech delay, clumsiness, frequent falls, progressive ataxia, dystonia, dysarthria, dysphagia, seizures (partial/generalized), gelastic cataplexy, VSGP, and hearing loss. 8

**Table 1:** Systemic and neurological manifestations according to the age group

Age group	Systemic manifestations	Neurological/psychiatric manifestations
Pre-/perinatal (< 2 months)	Fetal ascites/hydrops Hepatosplenomegaly Prolonged neonatal jaundice/cholestasis Thrombocytopenia Pulmonary disease Liver failure Failure to thrive	Hypotonia
Early-infantile (2 months to < 2 years)	Hepatosplenomegaly or splenomegaly (isolated or with neurological manifestations) Prolonged neonatal jaundice/cholestasis	Central hypotonia Delayed developmental motor milestones, speech delay Dysphagia, spasticity VSGP Ataxia
Late-infantile (2 to < 6 years)	Hepatosplenomegaly or splenomegaly (isolated or with neurological manifestations) History of prolonged neonatal jaundice/cholestasis	Developmental delay/regression, speech delay Clumsiness Frequent falls Progressive ataxia, dystonia, dysarthria, dysphagia Seizures (partial/generalized) Gelastic Cataplexy VSGP Hearing loss
Juvenile (6 to 15 years)	Hepatosplenomegaly or splenomegaly (isolated or with neurological manifestations; often not present)	Poor school performance, learning disability Loss of language skill Frequent falls, clumsiness Progressive ataxia, dysarthria, dystonia, dysmetria, dyskinesia, dysphagia VSGP Gelastic cataplexy Seizures Behavioral problems
Adolescent/adult (> 15 years)	Splenomegaly (often not present; isolated in very rare cases)	Cognitive decline, dementia, learning disability Psychiatric signs: Schizophrenia-like (atypical psychosis), depression. Clumsiness, progressive motor symptoms, tremor, ataxia, dystonia/dyskinesia, dysarthria, dysphagia VSGP

Abbreviation: VSGP, vertical supranuclear gaze palsy.

Adapted from: Geberhiwot et al. Consensus clinical management guidelines for Niemann-Pick disease type C. Orphanet J Rare Dis. 2018;13(1):50. 8

Regarding juvenile patients, manifestations include hepatosplenomegaly or splenomegaly (isolated or with neurological manifestations; often not present), poor school performance, learning disability, loss of language skills, frequent falls, clumsiness, progressive ataxia, dysarthria, dystonia, dysmetria, dyskinesia, dysphagia, VSGP, gelastic cataplexy, seizures, and behavioral problems. Finally, adolescent/adult patients may present splenomegaly (often not present; isolated in very rare cases), cognitive decline, dementia, and learning disability. 8

Despite the most frequently observed clinical characteristics, NPC may also present through atypical manifestations, as a fatal systemic perinatal form (characterized as intrauterine hydrops or early liver, multi-organ, or respiratory failure) and an initial systemic disease only (characterized as infants and children with a variable latency before the onset of neurological manifestations).

Lorenzoni et al. (2014) analyzed 5 NPC disease cases in Brazil. Regarding clinical presentation, all patients had vertical supranuclear gaze palsy, cerebellar ataxia, dementia, dystonia, and dysarthria. Other clinical characteristics observed were psychiatric disorders (n = 4), epilepsy (n = 2), dysphagia (n = 4), dysphonia (n = 4), weakness (n = 4), hepatosplenomegaly (n = 3), cataplexy (n = 2), cholestasis (n = 1), and deafness (n = 1). 11

#### *Diagnostic tests*

**Figure 2:**
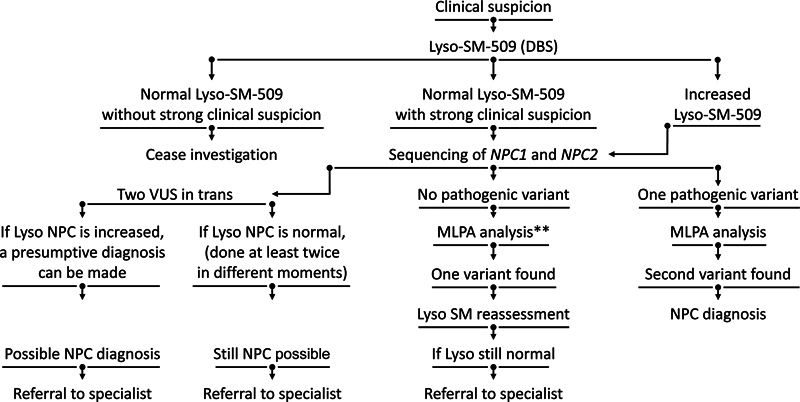
Flowchart for diagnostic confirmation, considering the Brazilian scenario. Abbreviations: Lyso-SM-509: Lyso-sphingomyelin 509; MLPA: multiple ligation dependent probe amplification; NPC: Niemann-Pick Type C; VUS: variant of uncertain significance. Note: **Other comprehensive molecular techniques may be required.

Once clinical manifestations are observed, and the suspects NPC disease, several diagnostic tests may be performed. 12 13 14 15 Table 2 shows all the existent options and comments about each one. Figure 2 presents the flowchart for diagnostic confirmation suggested by the expert panel, considering the Brazilian scenario of access to health resources. It is worthy of note that sequencing of only exons and exon–intron boundaries does not allow the identification of deep intronic variations, variations in regulatory regions, or large structural variants. 8 As genomic deletions and deep intronic variations are not uncommon in NPC disease, more detailed molecular characterization might be necessary in establishing a definite diagnosis in some challenging and complex cases. 14 Thus, in certain cases in which no pathogenic variations are found (or just one is identified), there will be the need for sequencing of promoters and deeper intronic regions, and more comprehensive molecular techniques should be used, such as multiplex ligation-dependent probe amplification (MLPA) and quantitative polymerase chain reaction (PCR) to detect genomic rearrangements (exonic or whole gene deletions). 16 Nevertheless, variations resulting in aberrant splicing may also require assessment of mRNA degradation (inhibition of nonsense-mediated mRNA decay processes) to be detected, and referral for an expert center is highly advised for complementary investigation. 8

**Table 2:** Complementary exams and best request moment

Exams	Options	Comments
Lyso-sphingolipids	Lyso-sphingomyelin (lyso-SM); Lyso-sphingomyelin 509 (lyso-SM 509)	Lyso-SM and Lyso-SM-509 are usually increased in NPC and also in ASMD and some other LSDs; the ratio Lyso-SM/Lyso-SM 509 usually helps to discriminates NPC from other LSDs. These measurements are becoming increasingly popular as they could be performed in dried blood spots.
Molecular genetics	*NPC1* and *NPC2* gene sequencing MLPA	Important to identify the underlying variations and confim NPC diagnosis. When two pathogenic variations are not identified, can help to unravel the molecular pathology.
Oxysterols	Cholestan-3β; 5α, 6β-triol (C-triol); 7- ketocholesterol (7-KC)	Useful screening methods, but the fact that are not usually available in DBS and the limited stability of the samples preclude its wide use; false positive can be found in cholestatic infants.
Bile acids	3β- trihydroxy-cholanoylglycine; 5α- trihydroxy-cholanoylglycine; 6β-trihydroxy-cholanoylglycine	Not widely available.
Filipin test	Observation of fluorescente after staining of live fibroblasts in culture	May be useful in challenging cases, but requires the collection of a skin biopsy and cultivation of fibroblasts; very limited availability.

Abbreviations: ASMD, acid sphingomyelinase deficiency; DBS, dried blood spots; LSDs, lysosomal diseases; MLPA, multiple ligation dependent probe amplification; NPC, Niemann-Pick disease type C.

Ribas et al. (2016) analyzed the cholestane-3β,5α,6β-triol levels, chitotriosidase activity, filipin staining, and *NPC* gene variations in Brazilian patients with clinical suspicion of NPC disease. Patients with high concentrations of 3β,5α,6β-triol also had high chitotriosidase activity, most of which were positive in the filipin test. These results demonstrate the sensitivity and specificity of different measures. 17

Regarding neuroimaging, the expert panel highlighted the difficulty of access in the national scenario. Even so, if it is necessary to choose only one neuroimaging exam, the brain magnetic resonance imaging (MRI) should be chosen instead of computed tomography (CT).

### Treatment

#### *Supportive care*

Niemann-Pick type-C disease is not a curable disease; however, it is a manageable condition. Thus, all patients must receive supportive care immediately after NPC diagnosis, regardless of symptoms and/or manifestations, to reduce the disease impact. In Table 3 , suggestions of therapies are proposed, according to Geberhiwot et al. (2018). 8 It is important to highlight the need for diet and lifestyle modifications to optimize stool consistency and avoid fecal impaction, incontinence, or diarrhea, since these are associated eith miglustat treatment.

**Table 3:** Supportive care and potential benefits

Supportive care	Potential benefits
Structured and personalized rehabilitation program to provide appropriate walking/mobility aids and ankle-foot orthotics	Maintain optimal mobility and reduce falls.
Speech and language therapist, dietician instructions	Individuals with dysphagia may benefit from instruction in dietary modification and compensatory postures. Provision of education for patients' families about the eventual need for assisted feeding.
Baclofen, tizanidine, benzodiazepines, dantrolene sodium, and botulinum toxin injections	Such strategies may be useful when non-pharmacological agents are unsuccessful.
Diet and lifestyle modifications	To optimize stool consistency and avoid fecal impaction or incontinence or diarrhea.
Protriptyline, other tricyclic agents, or modafinil	Useful to manage cataplexy.
Antiepileptic drugs	Useful to manage seizures according to the type of crisis. Valproate concomitant use with miglustat must be carefully considered due to thrombocytopenia risk.
Hyoscine hydrobromide transdermal patches; glycopyrronium orally, subcutaneously, or via a gastrostomy and small doses of orally administered atropine, or parotid/submandibular glandular injections of botulinum toxin	Useful to manage hypersalivation/drooling.
Annual hearing assessment and devices	Improve general communication.

#### *Disease-modifying therapy*

Currently, only one disease-modifying therapy for NPC disease is available worldwide. Although miglustat (approved by the Brazilian Health Regulatory Agency [Agência Nacional de Vigilância Sanitária – ANVISA, in Portuguese] in 2019) is not reimbursed for NPC disease treatment by the Brazilian public healthcare system, 15 expert panel recommends that all NPC patients, but the ones with end-stage neurological disease or with only visceral symptoms, are suitable for such therapy. This recommendation is supported by the international consensus report published in 2018. 8 Miglustat must be initiated at the first neurological or psychiatric sign, although the literature is still controversial regarding its benefit.

Freihuber et al. (2023) described a retrospective cohort of patients with NPC disease from France and did not observe improvement on long-term neurodevelopmental or overall survival comparing treated and untreated individuals. 18 Patterson et al. (2020) assessed the long-term survival of a large cohort of patients with NPC disease treated with miglustat, from 5 countries (Brazil, Czech Republic, France, United Kingdom, and the United States). Contrastingly, the results showed that the treatment promoted a significant decrease on mortality risk for both study groups, the one classified by age of neurological onset (hazard ratio [HR]: 0.51; 95% confidence interval [95%CI]: 0.335–0.777; *p* < 0.001), and the one grouped by age of NPC diagnosis (HR: 0.44; 95%CI: 0.293–0.664; *p* < 0.001), which was consistent in all age of disease onset groups. 19 Nadjar et al. (2018) assessed the long-term miglustat treatment effect in a cohort of patients with disease onset at adolescent/adult age and reported that individuals receiving the therapeutic strategy for a period > 2 years had a significant increase in overall survival and slower disease progression than untreated ones or those treated for < 2 years. 20

Still, regarding the benefit of miglustat for patients with NPC disease, Lorenzoni et al. (2014) reported a series of 4 Brazilian patients that used the drug. The authors reported that a clear benefit was observed in at least 1 patient; however, they did not provide further details on the response to treatment of the other 3 patients. Considering safety aspects, only 1 patient had the miglustat dosage decreased due to adverse events (gastrointestinal issues). 11 Finally, Santos et al. (2008) reported a case of a Brazilian child treated with miglustat. Improvements in several aspects such as speech, ptosis, ophthalmoplegia, ataxia, hypotonia, and seizures were observed after 12 months, with a well-tolerated profile. 21

In a scenario in which a scarcity of financial resources is observed, understanding the relationship between clinical benefits and the cost of health technologies is of major importance. Only one publication is available in the current literature assessing this outcome, to the best of our knowledge. Considering the perspective of the Serbian Republic Health Insurance Fund and an 8-year time horizon, miglustat was not a cost-effective option in comparison to symptomatic therapy for the treatment of NPC disease. The authors suggest that given the efficacy of miglustat, a lower price could change this scenario. 22 In addition, further analyses addressing the perspective of other countries, such as Brazil, are still needed.

Other therapeutic strategies are under investigation, such as arimoclomol (NCT02612129) and VTS-270 (2-hydroxypropyl-β-cyclodextrin; NCT03879655, NCT02534844, NCT04958642, NCT03471143). Arimoclomol is a molecule that amplifies the natural response to cellular stress, more directly through facilitation of the lysosomal function, and avoiding abnormal cell apoptosis. Preliminary results of the clinical trial demonstrated that the treatment significantly decreased the risk of disease progression in 12 months compared to placebo and was used in association with miglustat to stabilize disease severity; it was recently approved by FDA for NPC treatment in USA. 23 Treatment with VTS-270 has demonstrated slow disease progression at 18 and 36 months; however, there are different ongoing trials to assess its safety and efficacy. 5 24 25 26 A recent double-blind, placebo-controlled, crossover trial evaluated the effect of N-acetyl-dl-leucine (NALL) in patients with NPC. N-acetyl-dl-leucine is the l-enantiomer of N-acetyl-dl-leucine, a modified acetylate derivative of a natural essential amino acid leucine. The authors evaluated 60 patients older than 4 years of age with mild or moderate symptoms. The primary endpoint of the study was improvement in the total score of the Scale for the Assessment and Rating of Ataxia (SARA), which showed more progress with NALL than with placebo. 27

#### *Patient follow-up*

Considering the progressive course of the disease, physicians must follow-up patients with NPC disease on a regular basis. Table 4 presents the main concenrs, and which actions should be taken to promote proper care. Such practices are supported by the international consensus report published in 2018. 8

**Table 4:** Concerns and proper follow-up actions

Concern	Actions
Growth and developmental delay	Regular assessment of height, weight, and head circumference. Regular assessment of developmental progress, using age-appropriate instruments.
Mobility	Regular assessment of mobility, balance, core stability, trunk control, spasticity, foot posture, and strength.
Swallowing and diet	Regular assessment of swallowing by a speech and language therapist. Regular nutritional review by a dietician. Videofluoroscopic swallow study every 2 years.
Speech	Regular assessment of communication by a speech and language therapist.
Dystonia	Regular assessment of possible dystonia signs.
Spasticity	Regular assessment of spasticity and incipient or established contracture levels.
Cataplexy	Regular assessment of possible cataplexy signs.
Seizure	Regular assessment on seizure development and frequency. Electroencephalogram performance when it occurs, and annually or in case of refractory seizures.
Mental wellbeing (mood disorders and psychoses)	Patients shall be referred to clinical psychology/psychiatric team as appropriate, at any sign of mental disorders.
Sialorrhea	Regular assessment of salivation.
Hearing	Annual assessment of hearing.

In conclusion, NPC is a rare disease with scarce information on the Brazilian scenario. In this context, the expert panel provided an objective basis of recommendations on disease diagnosis, management, and follow-up in the country. The authors hope that this manuscript helps clinicians identify and adequately treat and follow-up NPC patients in Brazil.
